# Efficacy of zoledronic acid for chronic low back pain associated with Modic changes in magnetic resonance imaging

**DOI:** 10.1186/1471-2474-15-64

**Published:** 2014-03-04

**Authors:** Katri Koivisto, Eero Kyllönen, Marianne Haapea, Jaakko Niinimäki, Kaj Sundqvist, Timo Pehkonen, Seppo Seitsalo, Osmo Tervonen, Jaro Karppinen

**Affiliations:** 1 Medical Research Center Oulu, Oulu University Hospital and University of Oulu, Oulu, Finland; 2 Institute of Diagnostics, Department of Diagnostic Radiology, Oulu University Hospital, Oulu, Finland; 3 Department of Psychiatry, Oulu University Hospital, Oulu, Finland; 4 Rehabilitation Unit, Oulu Healthcare Centre, Oulu, Finland; 5 ORTON Orthopaedic Hospital, Helsinki, Finland; 6 Health and Work Ability, and Disability Prevention Centre, Finnish Institute of Occupational Health, Oulu, Finland; 7 Institute of Clinical Medicine, Department of Physical and Rehabilitation Medicine, University of Oulu, PL 5000, 90014 Oulu, Finland

**Keywords:** Low back pain, Magnetic resonance imaging, Modic changes, Randomized trial, Zoledronic acid

## Abstract

**Background:**

Modic changes (MC) are associated with low back pain (LBP), but effective treatments are lacking. The aim of this randomized, placebo-controlled, double-blinded trial was to evaluate the efficacy of zoledronic acid (ZA) for chronic LBP among patients with MC in magnetic resonance imaging (MRI).

**Methods:**

Inclusion criteria were LBP lasting ≥3 months, with an intensity of ≥6 on a 10-cm VAS or an Oswestry Disability Index (ODI) of ≥30%, and MC in MRI. Patients were randomized into single intravenous infusion of ZA 5 mg (n = 20), or placebo (n = 20) groups. The primary outcome was LBP intensity, secondary outcomes leg pain intensity, ODI, health-related quality of life (RAND-36), lumbar flexibility, sick leaves and use of pain medication. The treatment differences at one month and one year were analysed using ANCOVA with adjustment for the baseline score.

**Results:**

The mean difference (MD) between the groups in the primary outcome, intensity of LBP, was 1.4 (95% confidence intervals (CI) 0.01 to 2.9) in favour of ZA at one month. We observed no significant between-group difference in the intensity of LBP at one year (MD 0.7; 95% CI −1.0 to 2.4) or in secondary outcomes at any time point except that 20% of patients in the ZA group used non-steroidal anti-inflammatory drugs at one year compared to 60% in the placebo group (*P* = 0.022). Acute phase reactions (fever, flu-like symptoms, arthralgia) emerged in 95% of the patients in the ZA group, compared to 35% in the placebo group.

**Conclusions:**

ZA was effective in reducing the intensity of LBP in the short term and in reducing the use of NSAIDs within the time span of one year among patients with chronic LBP and MC confirmed in MRI. Although the results seem encouraging, larger studies are required to analyse the effectiveness and safety of ZA for patients with MC.

**Trial registration:**

ClinicalTrial.gov identifier NCT01330238.

## Background

Modic changes (MC) are pathological vertebral endplate and bone marrow changes visible in magnetic resonance imaging (MRI). Three different types of MC have been described; Type I (M1) lesions, considered to be the earliest and the most active stage in the process of MC evolution, are associated with vascular granulation tissue within the subchondral bone, whereas Type II (M2) lesions reflect fatty replacement of the red bone marrow [1]. The presence of mixed-type MC such as I/II (M1/2) has also been reported [2,3]. These are thought to reflect the conversion of MC from one type to another, representing different stages of the same pathological process [3-6].

MC are considered clinically relevant due to their association with chronic low back pain (LBP) [7-10]. This association was also found in a systematic literature review [11]. In general, M1 changes have been more frequently reported as being related to LBP than other MC types [7,9,12,13]. Moreover, the persistence of the M1 component correlates with persistence of symptoms [13]. However, recently the clinical relevance of MC has been questioned, as in prospective studies, MC were not indicated as having any prognostic role in future low back symptoms [14,15].

A limited number of therapeutic options have been evaluated for MC, as only two randomized trials on the treatment of MC have been published so far: a 100-day amoxicillin-clavulanate treatment was reported to have induced a marked improvement in LBP in chronic LBP patients with a M1 change after disc herniation [16], whereas another trial found no difference on the effects of rest and exercise on LBP with MC [17]. Bisphosphonates are considered a potential treatment option for MC as bone marrow lesions are less commonly observed in patients using alendronate [18]. Zoledronic acid (ZA) is a potent bisphosphonate, which can be administered intravenously once a year and has been shown to suppress osteoclast recruitment, differentiation and function, as well as promoting apoptosis [19]. ZA has been shown to reduce the progression of bone oedema in MRI with concordant improvement in clinical measures of disease activity among patients with psoriatic arthritis [19], and improvement in knee symptoms and bone marrow lesion size among patients with knee osteoarthritis [20]. The objective of our study was to evaluate the efficacy of a single intravenous infusion of 5 mg ZA in comparison with intravenous placebo infusion among patients with chronic LBP and MC in MRI.

## Methods

### Study design and selection of patients

This study was an investigator-initiated, single-centre, double-blinded, randomized, placebo-controlled clinical trial. Enrolled patients were referred from primary health care units to Oulu University Hospital, a tertiary care unit, where they were screened for eligibility by the principal investigator (KK). Inclusion criteria were low back symptoms for at least three months, an LBP intensity of at least six (6) on a 10-cm Visual Analog Scale (VAS) or an Oswestry Disability Index (ODI) of at least 30% [21], and an M1, mixed M1/2 or M2 in MRI performed within six months at most prior to enrolment. MRI scans were classified as previously described [9]; M1 lesions showing low signal intensity (SI) on T1-weighted (T1W) and high SI on T2-weighted (T2W) images, M2 lesions showing high SI on both T1W and T2W, and M3 showing low SI on both T1W and T2W.

The exclusion criteria included renal impairment with reduced creatinine clearance defined as an estimated glomerular filtration rate (eGFR) below 40 ml/min, hypocalcaemia, known hypersensitivity to ZA or other bisphosphonates or ingredients of the infusion product, the presence of red flags, nerve root entrapment and willingness for early retirement. Premenopausal women of childbearing potential were also excluded. Blood samples were taken prior to the infusion to assess the serum concentration of calcium and creatinine. The clinical examination included medical history and clinical assessment of lumbar flexibility, tendon signs, and motor and sensory testing.

The Oulu University Hospital ethics committee approved the study protocol. All patients provided written informed consent before any study-specific procedures were performed. This study was registered (ClinicalTrials.gov, unique identifier NCT01330238) prior to the initiation of enrolment and was conducted in accordance with the principles of the Declaration of Helsinki.

### Treatment intervention

Participants were recruited between November 2008 and March 2011. After confirmation of eligibility patients were randomized to receive a single intravenous infusion of 5 mg ZA in 100 ml saline (n = 20) or 100 ml saline as placebo (n = 20) over a 15-minute period. The principal investigator (KK) administered the infusions, assisted by a nurse.

Before administration of the infusion, all patients received oral ibuprofen 600 mg or paracetamol 1 g as prophylaxis for potential acute phase reactions such as flu-like symptoms, headache or fever. Patients were advised to use the same medication should post-dose symptoms appear. They all also received 100 000 units of Vitamin D (Vigantol^®^) orally to prevent hypocalcaemia. Information on use of the concomitant medication and hospital admissions were recorded. Blood samples were taken for the assessment of safety, inflammatory mediators and markers of bone turnover at baseline, one month and one year.

### Treatment assignment

A master randomization list was generated by a computer in blocks of eight, containing four placebo and four ZA allocations in random order. Patients were assigned a unique randomization number according to the order of inclusion. Patients, the principal investigator performing the screening and follow-up assessments, the nurse, the radiologist evaluating the MRI scans, and the statistician performing the analysis were blinded to the treatment allocation. The ZA and placebo were supplied in identical bottles by Novartis Pharma, Basel, Switzerland, to a pharmacist who prepared the intravenous solutions according to the allocation list and supplied the solution without revealing the treatment code. The treatment allocation was concealed in sealed envelopes until completion of the one-year follow-up of the last patient and the codes were opened only after the statistical analysis.

### Outcome measures

Clinical assessments were performed 14 days before enrolment (screening visit), and follow-up visits at one month and one year after the infusion. The primary outcome was the change in the intensity of LBP on VAS. Secondary outcomes included leg pain intensity, ODI, health-related quality of life assessed with RAND-36 [22], patient-reported sick leaves and lumbar flexibility. These outcome measures were assessed at baseline and at each follow-up. Lumbar flexibility was evaluated using the fingers-to-floor and trunk side bending measures (in cm). Pain medication use was inquired about during the follow-up visits.

### Safety parameters

The occurrence of any adverse effects was observed during the infusion and inquired about at each of the follow-up visits.

### Statistical analysis

Baseline characteristics of demographics and symptoms were described using mean values (with standard deviation, SD), frequencies (with proportions) or median values (with interquartile range). Treatment effects at one month and one year were analysed by comparing the change in the outcomes of the treatment groups (mean, 95% confidence interval (CI)) by using the independent samples t-test (crude p-values for group differences), and analysis of covariance (ANCOVA) with adjustment for the baseline score. We also adjusted the treatment difference for age and gender but the point estimates did not change considerably - only the confidence intervals widened. RAND-36 was analysed by using the sum of all items (total), and separate sums of physical and psychiatric items. The sums were standardized to follow normal distribution with a mean of 50 and standard deviation of 10 (N(50,10)). We also analysed the percentage of patients undergoing a 20% relative improvement and the proportion of patients reaching a VAS score of 40 or less in the primary outcome, patient acceptable symptom state (PASS) as recommended by Tubach et al. [23]. We used IBM SPSS Statistics 21.0 (IBM Corp., Armonk, NY) for statistical analyses, and considered p-values of <0.05 statistically significant.

## Results

### The study population

**Figure 1:**
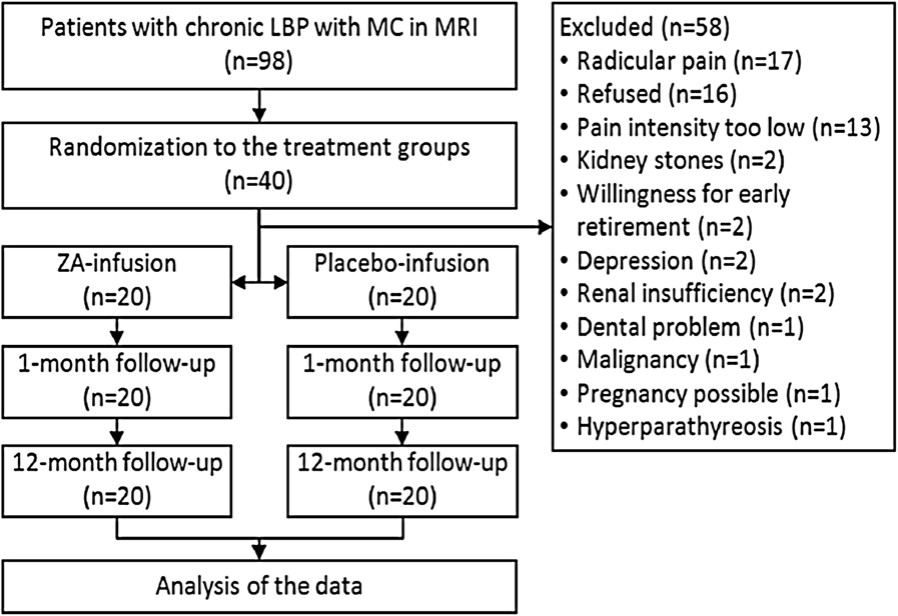
Study flowchart.

A total of 98 patients were screened for the study. More than half of them, 58 patients, were excluded as they did not meet the inclusion criteria (n = 35), refused to participate (n = 16), or had kidney stones (n = 2), depression (n = 2), dental problems (n = 1), malignancy (n = 1) or hyperparathyreosis (n = 1). All 40 enrolled, eligible patients completed the one-year follow-up (Figure 1).

The clinical characteristics of study participants at baseline are displayed in Table 1. The mean LBP duration was 293 days, initial LBP intensity on VAS 6.7, leg pain on VAS 2.9 and the ODI score was 32%. Altogether 19 patients in the ZA group and 18 in the placebo group had a mixed-type M1/2 lesion. MC were most commonly (70%) situated at L4/5 or L5/S1. The ZA and placebo groups were similar as regards the demographic and background characteristics of all patients at baseline, although there were numerically more men (15 vs. 11) in the ZA group than in the placebo group (Table 1).

**Table 1:** Baseline characteristics of study population according to treatment group

**Characteristics**	**Zoledronic acid**	**Placebo**
	**n = 20**	**n = 20**
Sex, n (%) men	15 (75)	11 (55)
Age, mean (SD) years	49 (9.3)	51 (7.3)
Smoking, n (%) regular smokers*	5 (25)	6 (30)
BMI, mean (SD) kg/m	26 (3.3)	27 (3.2)
Workload, n (%)		
-Sedentary work with limited walking	4 (20)	4 (22)
-Fairly light work with considerable walking but no lifting or carrying heavy objects	4 (20)	3 (17)
-Fairly strenuous work with walking and lifting heavy objects or climbing stairs or uphil	8 (40)	6 (33)
-Very strenuous work with lifting or carrying heavy objects such as shovelling, digging or hammering	4 (20)	5 (28)
Type of worst MC-lesion**, n		
Type I	1	1
Type I/II	19	18
Type II	0	1
MC at two or more levels, n (%)	7 (35)	4 (20)
Levels of MC, n		
L2/3	4	0
L3/4	3	5
L4/5	6	5
L5/S1	7	10
Duration of LBP, median (IQ range) days	330 (200, 365)	315 (270, 365)
Intensity of LBP, mean (SD)***	6.6 (1.4)	6.8 (1.6)
Duration of leg pain, median (IQ range) days	50 (0, 100)	36 (0, 160)
Intensity of leg pain, mean (SD)***	3.0 (3.1)	2.9 (2.3)
Oswestry Disability Index, %, Mean (SD)	30 (11)	35 (10)
Duration of sick leave during the past year, median (IQ range) days	14 (0, 48)	18 (1, 181)
RAND-36, mean (SD)	50 (8)	50 (7)
RAND-36 physical component, mean (SD)	51 (8)	49 (8)
RAND-36 mental component, mean (SD)	51 (8)	49 (9)

BMI = Body Mass Index, MC = Modic change, LBP = low back pain, SD = standard deviation, IQ = inter-quartile.

*Smoking at least one cigarette/day.

**If different types of MC at two or more levels, classification is based on the assumed severity of the type, i.e. Type I > mixed Type I/II > Type II.

***Assessed using a 10-cm Visual Analogue Scale (VAS).

### Treatment differences

The mean difference (MD) between the treatment groups in the primary outcome, intensity of LBP, significantly favoured ZA at one month (MD 1.4; 95% CI 0.01 to 2.9) while at one year no significant difference was observed (MD 0.7; 95% CI −1.0 to 2.4; Table 2). The proportion of patients with at least 20% improvement in intensity of LBP and PASS both favoured the ZA treatment at one month: ZA 55% vs. placebo 25% (p = 0.105) and ZA 50% vs. placebo 20% (p = 0.096), respectively.

**Table 2:** Low back symptoms and lumbar flexibility at baseline, one month and 12 months according to treatment group and between group comparisons of difference from baseline to one month and 12 months

	**Mean (SD) original values**		**Mean (SD) change**		**Unadjusted analyses**		**Adjusted analyses**	
	**ZA**	**Placebo**	**ZA**	**Placebo**	**Difference**	**P**	**Difference**	P*
	**n = 20**	**n = 20**			**(95% CI)**		**(95% CI)**	
Intensity of LBP								
Baseline	6.6 (1.4)	6.8 (1.6)						
1 month	4.3 (2.3)	5.8 (2.2)	−2.2 (2.7)	−0.9 (2.1)	1.3 (−0.2 to 2.8)	0.097	1.4 (0.01 to 2.9)	0.049
12 months	3.8 (2.5)	4.6 (2.9)	−2.8 (2.9)	−2.2 (2.5)	0.6 (−1.1 to 2.4)	0.474	0.7 (−1.0 to 2.4)	0.387
Intensity of leg pain^a^								
Baseline	3.0 (3.1)	2.9 (2.3)						
1 month	2.0 (2.3)	3.0 (2.4)	−0.6 (2.4)	0.1 (2.6)	0.8 (−0.9 to 2.4)	0.367	0.8 (−0.6 to 2.2)	0.237
12 months	2.1 (2.8)	2.7 (2.6)	−0.9 (3.4)	−0.3 (3.0)	0.6 (−1.5 to 2.7)	0.573	0.5 (−1.3 to 2.2)	0.573
Oswestry disability index, %								
Baseline	30 (11)	35 (10)						
1 month	24 (10)	33 (13)	−5.9 (11)	−1.7 (9.7)	4.3 (−2.5 to 11)	0.212	6.0 (−0.6 to 13)	0.071
12 months	25 (13)	33 (15)	−5.0 (15)	−1.9 (12)	3.1 (−5.6 to 12)	0.475	5.1 (−3.4 to 14)	0.231
Fingers-to-floor, cm								
Baseline	23 (19)	19 (18)						
1 month	17 (17)	19 (17)	−5.1 (20)	−0.1 (8.3)	5.0 (−4.8 to 15)	0.306	3.6 (−5.0 to 12)	0.403
12 months	16 (16)	20 (19)	−6.3 (23)	0.9 (11)	7.1 (−4.3 to 18)	0.215	5.3 (−4.5 to 15)	0.277
Sidebending to right, cm								
Baseline	14.1 (4.9)	13.8 (7.2)						
1 month	15.7 (5.9)	13.3 (6.9)	1.5 (4.7)	−0.5 (2.2)	−2.0 (−4.3 to 0.4)	0.101	−2.0 (−4.4 to 0.3)	0.087
12 months	15.7 (5.6)	13.8 (6.5)	1.6 (4.8)	−0.1 (3.5)	−1.6 (−4.3 to 1.1)	0.227	−1.7 (−4.2 to 0.8)	0.180
Sidebending to left, cm								
Baseline	15.0 (5.4)	13.3 (5.5)						
1 month	16.1 (5.3)	12.8 (5.9)	1.1 (3.0)	−0.5 (2.2)	−1.5 (−3.2 to 0.1)	0.072	−1.7 (−3.4 to 0.0)	0.051
12 months	16.2 (6.7)	13.7 (5.7)	1.2 (5.3)	0.5 (3.2)	−0.7 (−3.5 to 2.1)	0.601	−1.0 (−3.8 to 1.8)	0.458

SD = standard deviation, CI = confidence interval, ZA = zoledronic acid, LBP = low back pain.

*ANCOVA: Difference between follow-up and baseline, treatment effect adjusted for baseline value.

^a^One subject missing at baseline in placebo group and in ZA group, and one subject at 1 month in ZA group.

Of the secondary outcomes, the improvement in ODI, favored non-significantly ZA at 1 month, the adjusted between-group difference being 6.0% (95% CI −0.6 to 13), but not at one year (Table 2). Similarly, side bending (to right and left) non-significantly favoured the ZA treatment at one month but not at one year (Table 2). We observed no differences between the treatment groups at any time point in leg pain intensity (Table 2), total RAND-36, or in the physical and mental components of RAND-36 (Table 3).

**Table 3:** Health-related quality of life assessed using RAND-36 at baseline, one month and 12 months according to treatment group and between group comparisons of difference from baseline to one month and 12 months

	**Mean (SD) original values**		**Mean (SD) change**		**Unadjusted analyses**		**Adjusted analyses**	
	**ZA**	**Placebo**	**ZA**	**Placebo**	**Difference**	**P**	**Difference**	P*
	**n = 20**	**n = 20**			**(95% CI)**		**(95% CI)**	
Total RAND-36								
Baseline	50 (8)	50 (7)						
1 month	51 (8)	49 (8)	0.6 (6.4)	−0.6 (5.0)	1.2 (−3 to 5)	0.530	1.3 (−3 to 5)	0.477
12 months	51 (8)	49 (9)	1.0 (8.7)	−1.0 (5.9)	2.1 (−3 to 7)	0.378	2.2 (−2 to 7)	0.314
Physical component								
Baseline	52 (8)	48 (8)						
1 month	52 (9)	48 (8)	0.1 (8.6)	−0.1 (5.5)	0.3 (−4 to 5)	0.897	1.3 (−3 to 6)	0.554
12 months	52 (8)	48 (2)	0.3 (10)	−0.3 (6.5)	0.7 (−5 to 6)	0.808	2.1 (−3 to 7)	0.405
Mental component								
Baseline	49 (9)	51 (8)						
1 month	50 (9)	50 (9)	1.0 (6.1)	−1.0 (5.6)	2.0 (−2 to 6)	0.286	1.6 (−2 to 5)	0.396
12 months	51 (9)	49 (9)	1.8 (9.0)	−1.8 (6.7)	3.5 (−2 to 9)	0.167	2.7 (−2 to 7)	0.261

SD = standard deviation, CI = confidence interval, ZA = zoledronic acid.

*ANCOVA: Difference between follow-up and baseline, treatment effect adjusted for baseline value.

At baseline, there were no differences in self-reported use of non-steroidal anti-inflammatory drugs (NSAIDs) between the treatment groups, whereas at one year, only 20% of patients in the ZA group used NSAIDs versus 60% in the placebo group (*P* = 0.022). No significant differences were observed in patient-reported days of sick leave (data not shown).

### Safety parameters

Reported adverse events (AE) were common and occurred more frequently in the ZA group, especially immediately after the infusion. AEs were mostly mild in nature (Table 4). Despite prophylaxis, acute post-infusion phase reactions (fever, headache, myalgia, arthralgia, pain, nausea and flu-like symptoms) were observed in 19/20 patients in the ZA vs. 7/20 patients in the placebo group. As expected, the majority of the acute phase reactions were of mild to moderate severity as rated by the investigator and typically resolved within three days of onset. One event met the criteria for serious adverse effect (SAE) in the ZA group; a male patient had sinusitis requiring temporary hospitalization after the infusion.

**Table 4:** Adverse events

**Adverse events**	**ZA**	**Placebo**
	**n = 20**	**n = 20**
Participants with at least one adverse event	19 (95%)	7 (35%)
Acute phase reaction		
Flu like symptoms	2	3
Fever	19	1
Headache	6	1
Myalgia	15	4
Arthralgia	6	0
Abnormal blood results		
Elevated CRP	1	0
Serious adverse events		
Prevalence of at least one serious adverse event	1	0
At least one non-elective hospital admission	1	0
Death	0	0

ZA = zoledronic acid.

## Discussion

A single intravenous infusion of 5 mg ZA resulted in a greater improvement in LBP intensity at one month. Furthermore, the patients receiving ZA reported NSAID use at one year significantly less often than those in the placebo group. Overall, the improvements in most of the evaluated parameters were greater in the ZA group throughout the follow-up period. Adverse events were commonly observed in our study, but as expected, the reported events mostly consisted of mild to moderate acute phase reactions, as described in the literature [24,25].

The natural course of MC is not well known. Usually M1 lesions convert to M2 lesions with time [5], although small M1 lesions may also normalize [6]. According to the current view, the persistence of the M1 component correlates with persistence of symptoms [13,26]. We observed in another study population that symptoms persisted in almost one third of patients over a two-year follow-up, and that this persistence of symptoms was related to the persistence of the M1 component (Järvinen, unpublished observation). It is interesting to evaluate the course of symptoms in relation to changes in the M1 component on MRI in the current study population.

The current theories on the pathomechanisms of MC include discogenic inflammation [27] and low-grade bacterial infection [28]. The bacterial infection theory was supported by a recent demonstration of the presence of anaerobic bacteria in lumbar disc herniation in 80% of the new M1 changes [29], and by the positive results of a trial with antibiotic treatment [16]. On the other hand, in another Danish study, no anaerobic bacteria were found in biopsies from vertebrae with M1 lesions [30].

The suggested role of discogenic inflammation is based on the observation that the cartilaginous endplates of patients with M1 contained more tumour necrosis factor (TNF) immunoreactive cells than those of patients with M2 changes or with normal endplates [27]. Intradiscal glucocorticoid injection is therefore a logical treatment choice in cases of inflammation and consequently published case studies on intradiscal steroids into discs with M1 show improvement in symptoms in the short term [31,32] and even normalization of M1 changes in MRI [32]. Similarly, in some cases patients with M1 had a greater medium-term improvement in disability when treated with epidural steroid injections [33].

Segmental instability has also been claimed to cause MC [7]. Two small case studies among patients with chronic LBP showed that patients with M1 changes benefitted from instrumented fusion [4,34]. The presence of MC did not negatively influence the outcome of total lumbar disc replacement among patients with degenerative disc disease [35].

Bisphosphonates (BPs) are synthetic analogues of the endogenous bone mineralization regulators, pyrophosphates, and have shown to be potent inhibitors of osteoclast activity [36]. Nitrogen-containing bisphosphonates, such as ZA, inhibit farnesyl diphosphonate synthase and block prenylation of guanosine triphosphate-binding protein [37], control osteoblastic proliferation and differentiation [38], modulate osteoblast production of extracellular matrix proteins, regulate the secretion of several cytokines and growth factors, and enhance the proliferation and maturation of bone marrow stromal cells into the osteoblastic lineage [39]. Bisphosphonates not only inhibit osteoclasts; it has also been demonstrated that they suppress the secretion of proinflammatory cytokines such as interleukin 1 (IL-1), TNF-α and IL-6 [40]. Clodronate, a first-generation bisphosphonate, has shown to reduce synovial levels of prostaglandin E_2_[41]. The positive trends observed in our study may partially be due to the general ability of bisphosphonates to regulate bone turnover by suppressing osteoclast activity or to direct anti-inflammatory effects.

Previous studies have shown that RA patients treated with ZA presented fewer new bone-erosions and less frequently progressing bone oedema in MRI [42]. Among patients with psoriatic arthritis, ZA reduced the progression of bone oedema in MRI and clinical measures of disease activity, while ZA had no effect on the progression of erosions [19]. Similarly, pamindronate has been found to be effective in patients with ankylosing spondylitis refractory to NSAIDs [43]. Bisphosphonates are generally considered safe in various indications [37]. Our preliminary results are encouraging as, in addition to the significant effect in LBP intensity at one month, there was a noteworthy decrease in the use of NSAIDs in the ZA group at one year. The higher degree of NSAID use at one year in the placebo group probably dilutes the one-year treatment difference in the primary outcome. This is a clinically relevant finding as long-term chronic use of NSAIDs may increase the risk of gastrointestinal side-effects and cardiovascular events, which may be avoided with the use of ZA.

The strength of our study is the randomized trial design. Further strengths include complete follow-up with no drop-outs and 100% adherence as the medication was given intravenously. Moreover, intravenously administered bisphosphonates may have greater treatment effects than oral bisphosphonates [44].

However, some limitations of our study should also be discussed. The small sample size of this pilot study is inadequate to demonstrate clinically relevant changes in the outcomes. However, despite the small sample a favourable trend in the ZA group was observed for most of the outcomes. However, due to multiple testing, the significance levels of secondary outcomes must be interpreted with caution. We did no *a priori* power calculations due to the lack of any previous data on the efficacy of ZA in the studied indication. The patients were well informed of possible adverse effects; this may have contributed to a large amount of reports of acute phase reaction symptoms. Some of the main determinants of the risk of acute phase reactions include younger age and higher number of circulating inflammatory cytokines and lymphocytes such as gammadelta cells [24]. The patients, the study nurse, the medical team in charge of the patient, the physician performing the assessments and infusion, and the statistician performing the analyses were all blinded to the allocation. However, the high incidence of acute phase reaction symptoms in the ZA group may have revealed the concealment to some patients. Unfortunately, we did not evaluate the patients’ perception of the nature of the treatment they had received. Therefore pre-infusion prophylaxis treatment was assigned to all patients and the observed higher incidence of post-infusion symptoms was an expected finding in the ZA group. However, some patients in the control group also experienced acute phase reactions.

## Conclusions

To our knowledge, this is the first randomized controlled trial to investigate bisphosphonates in chronic, non-specific LBP. The improvement in the intensity of LBP was greater with a single intravenous infusion of 5 mg ZA compared to placebo at one month. We believe that ZA is an interesting therapeutic alternative for this common condition, which is difficult to treat effectively with conservative treatment approaches [17]. We acknowledge that ZA should only be reserved for patients with severe disabling LBP, with confirmed MC in MRI, and when symptoms are not adequately controlled with pain medication and physiotherapy. Although the results are encouraging, larger studies are required to prove the efficacy of ZA in patients with LBP due to MC.

## Competing interests

The authors declare that they have no competing interests.

## Authors’ contributions

All authors were involved in drafting the article or revising it critically and interpreting the results. KK wrote the first drafts of the manuscript with the guidance of JK. MH managed the data analyses. All authors approved the final version for publication.

## Pre-publication history

The pre-publication history for this paper can be accessed here:

http://www.biomedcentral.com/1471-2474/15/64/prepub
